# Locus Coeruleus Magnetic Resonance Imaging in Neurological Diseases

**DOI:** 10.1007/s11910-020-01087-7

**Published:** 2020-12-12

**Authors:** Alessandro Galgani, Francesco Lombardo, Daniele Della Latta, Nicola Martini, Ubaldo Bonuccelli, Francesco Fornai, Filippo Sean Giorgi

**Affiliations:** 1 Neurology Unit, Pisa University Hospital, Pisa, Italy; 2 U.O.C. “Risonanza Magnetica Specialistica e Neuroradiologia”, Fondazione “G. Monasterio”- National Research Council/Tuscany Region, Pisa, Italy; 3 Deep Health Unit, Fondazione “G. Monasterio”- National Research Council/Tuscany Region, Pisa, Italy; 4 Department of Translational Research and of New Surgical and Medical Technologies, University of Pisa, Pisa, Italy; 5 IRCCS Neuromed, Pozzilli, Italy

**Keywords:** Locus coeruleus, Magnetic resonance imaging, Neuromelanin, Noradrenaline, Alzheimer’s disease, Parkinson’s disease

## Abstract

**Purpose of Review:**

Locus coeruleus (LC) is the main noradrenergic nucleus of the brain, and its degeneration is considered to be key in the pathogenesis of neurodegenerative diseases. In the last 15 years,MRI has been used to assess LC in vivo, both in healthy subjects and in patients suffering from neurological disorders. In this review, we summarize the main findings of LC-MRI studies, interpreting them in light of preclinical and histopathological data, and discussing its potential role as diagnostic and experimental tool.

**Recent findings:**

LC-MRI findings were largely in agreement with neuropathological evidences; LC signal showed to be not significantly affected during normal aging and to correlate with cognitive performances. On the contrary, a marked reduction of LC signal was observed in patients suffering from neurodegenerative disorders, with specific features.

**Summary:**

LC-MRI is a promising tool, which may be used in the future to explore LC pathophysiology as well as an early biomarker for degenerative diseases.

## Introduction

The locus coeruleus (LC) is the main noradrenergic (NA) nucleus of the central nervous system and its fibers widely innervate cortical and subcortical structures [1]. LC contributes to several brain functions. In particular, it plays a key role in sleep-waking cycle [2], modulates the neuro-glial homeostasis [3], regulates neurovascular unit proper functioning [4], and is key in specific neuropsychological processes, such as novelty-oriented attention and learning [5].

A significant LC degeneration occurs in the early stages of some neurodegenerative disorders (NDD), such as Parkinson’s Disease (PD)(reviewed in [6]) and Alzheimer’s Disease (AD) [7]; this has been suggested to be not just a mere epiphenomenon but to play a key pathogenetic role. The mechanisms through which LC degenerates in PD and AD differ: in AD, it is related to progressive accumulation of Tau pathology, starting from hyperphosphorylated TAU (pTAU) up to neurofibrillary tangles (NFT) [8], while in PD, it is related to Lewy-body pathology (i.e., to alpha-synuclein accumulation) [9].

A large amount of histological data concerning LC involvement in NDD [8, 10••, 11–15] has been produced in the last decades. Conversely, its estimation in vivo in patients has been obtained only recently by magnetic resonance imaging (MRI) with LC-sensitive sequences (LC-MRI) [16].

The latter tool has been used ever since by several authors to estimate LC features in vivo in physiological and pathological conditions.

In the following paragraphs, we will briefly describe the anatomy of LC and the potential pathogenetic role of LC degeneration as assessed by models of AD and PD. Then, we will describe the state of the art of LC-MRI, both by summarizing the main MRI sequences used and by reviewing the available data in healthy subjects and in NDD, with particular attention on AD and parkinsonism. In Table 1, it is provided a list of LC-MRI studies which have been performed in the latter populations of subjects, together with an overview of their features.

**Table 1:** LC-MRI studies performed in healthy subjects and patients suffering from neurodegenerative diseases or other neuropsychiatric disorders

Authors and year	Population	MR scan	LC T1 -sequences	LC Parameter(s) assessed	Interpretation
Healthy subjects and normal aging					
Shibata et al. 2006	64 HC	3 T (GE)	FSE	Intensity	LC intensity increases until 5–6th decades of life, and then it decreases in the elderlies
Clewett et al. 2016	56 HC (33 Y, 23 E)	3 T (Siemens)	FSE	Intensity	LC intensity is higher in the elderlies and it positively correlates with cognitive reserve
Betts et al. 2017	82 HC (25 Y, 57 E)	3 T (Siemens)	FLASH	Intensity	LC intensity increases with aging, reaching its maximum in the elderlies
Mather et al. 2017	45 HC (27 Y, 18 E)	3 T (Siemens)	FSE	Intensity	LC intensity positively correlates with heart requency parasympathetic modulation, both in young and elderly subjects
Clewett et al. 2018	22 HC	3 T (Siemens)	FSE	Intensity	LC intensity positively correlates with cognitive performances (prioritized memory under arousal)
Hammerer et al. 2018	50 HC (28 Y, 22 E)	3 T (Siemens)	FLASH	Intensity, volume	In elderly subjects, LC intensity positively correlates with emotional memory performance
Dahl et al. 2019	294 HC (66 Y, 228 E)	3 T (Siemens)	TSE	Intensity	Intensity of LC the rostral third correlates with verbal memory performances in elderlies
Liu et al. 2019	605 HC	3 T (Siemens)	3D SPGR MT	Intensity, volume	LC intensity homogenously increases in young subjects, while intensity of LC rostral third decreases in the elderlies
Liu et al. 2020	613 HC	3 T (Siemens)	3D SPGR MT	Intensity	Intensity of LC rostral third correlates with global cognitive performances
Alzheimer’s disease					
Miyoshi et al. 2013	20 HC 6 ADD	3 T (GE)	FSE	Intensity	No difference between ADD and HC
Takahashi et al. 2015	26 HC 38 MCI 22 ADD	3 T (GE)	FSE	Intensity	LC intensity is significantly reduced in ADD and MCI when compared to HC; no significant differences between ADD and MCI
Dordevic et al. 2017	10 HC 10 AD	3 T (Siemens)	TSE	Intensity	LC intensity is lower in AD than HC
Betts et al. 2019	25 HC 21 SCI 16 MCI 11 ADD	3 T (Siemens)	FLASH	Intensity	LC intensity is significantly reduced in ADD when compared to HC; no differences considering MCI and SCI; in ADD, LC intensity correlated with Ab42/Ab40 ratio
Olivieri et al. 2019	17 HC 21 typical AD 16 atypical AD	3 T (Siemens)	TSE	Intensity	LC intensity is reduced both in typical and atypical AD when compared to HC; LC intensity correlates to memory performance in typical AD
Parkinson’s disease and other parkinsonism					
Sasaki et al. 2006	22 HC 17 PD	3 T (GE)	FSE	Intensity	LC intensity is lower in PD than HC
Matsuura et al. 2013	23 HC 32 PD 9 MSA	3 T (Siemens)	FSE	Intensity	LC intensity is reduced both in PD and MSA when compared to HC; no correlation with disease severity
Garcia-Lorenzo et al. 2013	19 HC 12 PD(RBD−) 24 PD (RBD+)	3 T (Siemens)	TSE	Intensity	LC intensity is significantly reduced in PD (RBD+) subjects compared to HC; no differences between PD (RBD−) and HC; LC intensity correlated with the degree of abnormal muscle activity during. REM sleep
Ohtsuka et al. 2014	22 HC 30 mild PD 31 severe PD	3 T (GE)	TSE	Intensity	LC intensity is significantly reduced in PD compared to HC; no correlation with disease severity
Castellanos et al. 2015	37 HC 23 sporadic PD 13 genetic PD	3 T (Siemens)	FSE	Volume	LC volume is significantly reduced both in sporadic and genetic PD compared to HC; no differences between sporadic and genetic PD
Ehrminger et al. 2016	21 HC 21 RBD	3 T (Siemens)	TSE	Intensity	LC intensity is significantly decreased in RBD when compared to HC
Isaias et al. 2016	18 HC 18 PD	3 T (Phillips)	FSE	Intensity	LC intensity is lower in PD and it correlates with the degree of SPECT DAT SCAN impairment
Schwarz et al. 2017	30 HC 39 PD	3 T (GE) (Phillips)	Inversion recovery T1-MT SE-MT	Volume	LC volume is reduced in PD when compared to HC
Sommerauer et al. 2018	12 HC 16 PD(RBD+) 14 PD(RBD-)	3 T (Siemens)	TSE	Intensity	LC intensity is reduced in PD when compared to HC and it is lower in PD (RBD+) than PD (RBD−). In RBD+ patients, LC intensity correlated with dysautonomic dysfunction and cognitive performances
Wang et al. 2018	28 HC 23 PD(DEP+) 28 PD(DEP−)	3 T (GE)	FSE	Intensity	Left LC intensity is reduced in PD when compared to HC and it is significantly lower in PD (DEP+) than in PD (DEP−)
Li et al. 2019	32 HC 48 PD-NCI 23 PD-MCI	3 T (GE)	FSE	Intensity	LC intensity is significantly reduced in PD-MCI when compared to HC and it is lower (even not significant) in PD-NCI than HC; LC intensity correlates with TMT scores
Other neurological disease and psychiatric disorders					
Shibata et al. 2007	20 HC 43 DEP	3 T (GE)	FSE	Intensity	Intensity of LC rostral third is significantly reduced in depression when compared to HC
Shibata et al. 2008	34 HC 20 SCHIZ 18 DEP	3 T (GE)	FSE	Intensity	LC intensity is significantly reduced in depression, while it is not affected by schizophrenia
Morris et al. 2020	14 HC 8 ANX 7 PTSD	7 T (Siemens)	TFL/MT	Intensity, Volume	LC volume is significantly increased in subjects suffering from anxiety disorders and PTSD; no significant alteration of LC intensity
Gollion et al. 2020	23 HC 23 MIG	3 T (Phillips)	SE	Intensity, Volume	LC-MRI parameters are not affected in patients suffering from migraine with aura

*AD*, Alzheimer’s disease; *ADD*, AD dementia; *ANX*, anxiety disorder; *DEP*, depression; *E*, elderly subjects; *FLASH*, fast low angle shot; *FSE*, fast spin echo; *GE*, general electrics; *HC*, healthy control; *LC*, locus coeruleus; *MCI*, mild cognitive impairment; *MIG*, migraine with aura; *MSA*, multisystemic atrophy; *MT*, magnetization transfer; *NCI*, non-cognitive impairment; *NM*, neuromelanin; *PD*, Parkinson’s disease; *PTSD*, post-traumatic stress disorder; *RBD*, REM behavioral disorder; *SCI*, subjective cognitive impairment; *SCHIZ*, schizophrenia; *SPGR*, spoiled gradient echo; *T*, Tesla; *TFL*, turbo FLASH; *TMT*, trail making test; *TSE*, turbo spin echo; *Y*, young subjects

### LC Anatomy

LC is the main source of NA in the brain. It is a tube-shaped and symmetric nucleus, placed in the pons, right below the floor of the fourth ventricle. According to Dahlström and Fuxe’s classification of brainstem nuclei [17], LC is also named as NA nucleus A6 and it is considered as a NA neuronal complex, together with its most rostral extension, the nucleus epi-coeruleus (A6e), and its most ventral one, the nucleus sub-coeruleus (A6sc); for the sake of simplicity, such a complex is often named just as LC [18].

LC is 12–17-mm-long and 2.5-mm width. Each LC includes approximately 12.000–60.000 NA cells [19, 20], which are rich in neuromelanin (NM) [21]. NM is a pigmented molecule which gives LC its characteristic color; it is a by-product of NA metabolism and a chelator of metal ions, and therefore it is thought to protect NA cells from oxidative damage caused by these ions within LC neurons [22]. It has been proposed that the combination of NM with ions and macromolecules (including proteins and lipids) within LC neurons contributes to its T1-shortening effects and can be visualized on T1-weighted MR images [23••] (but see also paragraph 1.3).

LC belongs to the so-called iso-dendritic core, a group of brainstem nuclei characterized by great convergence of afferent inputs and by sending diffuse projections throughout the whole brain [24]. LC neurons are organized according to a topographical distribution based on their specific projections targets: neurons projecting to cortical and subcortical structures (e.g., limbic lobe, basal forebrain, neocortex) are placed mainly in the rostral part of the nucleus, while neurons targeting the cerebellum and spinal cord are located in in the ventral and caudal part of LC [25].

### Potential Role of LC in the Pathophysiology in Neurodegenerative Disorders

An early impairment of LC occurs both in AD [8] and PD [9] (see also par. 4 and 5) and it is considered to be crucial in their pathogenesis for two main reasons; on the one hand, LC-NA system breakdown may hamper the maintenance of neuronal homeostasis [26, 27]; on the other, the diffuse and wide projections from LC may concur to the spreading of neurodegenerative process toward other brain structures [28].

In AD, several studies performed in experimental models have linked LC impairment with amyloid/tau pathology, neuroinflammation, and neurovascular disfunction [29–31]. In particular, selective LC lesion in AD transgenic mice causes a dramatic increase in amyloid plaques burden compared with LC-intact animals, in parallel with increased neuroinflammation [29]. In another AD mouse model, LC stimulation induces a significant reduction of cytokine levels in the cortex, together with increased microglial amyloid phagocytosis; on the contrary, LC lesion induces an increase in cytokine production and leads to aberrant microglial activation [30]. Thus, Heneka and colleagues concluded that LC-NA may be key in promoting amyloid clearance system and in modulating microglial activity [29, 30, 32], and several more recent experimental studies support this hypothesis [3, 33–35]. Furthermore, LC impairment has also been linked to AD vascular pathology by Kelly and colleagues who observed a significant exacerbation of microvascular injury and amyloid angiopathy, in AD transgenic mice submitted to selective LC lesion [31].

Apart from amyloid pathology and neuroinflammation, LC degeneration has been shown to be strongly related to tau pathology; as abovementioned, the first signs of AD-related pathological alterations (years before the first clinical symptoms and the occurrence of amyloid plaques) occur in LC and are represented by the accumulation of pTAU (also denominated “pre-tangle Tau”) [8]. Moreover, it has been observed that the NA metabolism by-product DOPEGAL, when produced in excess by NA neurons, is associated with tau aggregation [36], and that spreading of Tau pathology toward other brain structures might occur, at least in part, through LC efferents themselves [37]. Finally, it has been observed that LC lesion can worsen neurodegeneration and increase tau accumulation in AD transgenic mice [38].

In the last decades, several authors reproduced LC lesion also in animal models of PD (such as those in which substantia nigra (SN) pars compacta (SNpc) dopamine (DA) loss is induced by the neurotoxin MPTP or by substituted amphetamine administrations in rodents or primates) to assess whether LC degeneration might concur to PD pathogenesis rather than being just an epiphenomenon. In the early 1990s, Colpaert’s group showed that LC lesion could strongly potentiate nigrostriatal DA damage induced by MPTP in primates and in mice [39, 40]. Fornai and colleagues significantly extended these findings by showing that (a) LC lesion makes toxic for DA SNpc neurons otherwise sub-toxic doses of methamphetamine [41] and significantly potentiates nigrostriatal loss induced by systemic methamphetamine administration in mice and rats [42]; (b) the potentiating effects of LC lesion in these rodent models of PD are not due to a change of MPTP/MPP+ or methamphetamine pharmacokinetics, or to an impairment of DA loss recovery, but rather to a potentiation of the neurotoxic effects/mechanisms of MPTP/Methamphetamine themselves [41, 43]. Thus, such an effect of LC pre-lesion was obtained in different animal species [41, 44] and using different DA neurotoxins. This has been interpreted as a proof that the role of LC degeneration on the pathophysiololgy of nigrostriatal DA loss occurring in PD could be a sound phenomenon, which could be extended also to the human disease, according to the temporal sequence of events in which LC degeneration precedes DA loss [6].

### LC Assessment by MRI

LC can be visualized by MRI scan, profiting from specific MR sequences (see below, paragraph 1.4). However, its small size and the physiological inter-subjects variability [20] complicate the interpretation of LC-MRI findings. Also for these reasons, in recent years, specific studies have been designed to evaluate the correspondence between imaging and anatomical data, as well as to assess technique reliability.

In particular, in 2009, Keren and colleagues submitted 44 healthy subjects (HC), aged between 19 and 79 years, to 3 T (Tesla) LC-MRI and produced a probabilistic map of the LC. They found that the highest T1 signal could be observed in the rostral pons, and that the position and density of pontine T1-hyperintense voxels had a strong correspondence with LC placement data obtained in *post-mortem* specimens [45]. To further strengthen their observations, the same group performed a second study in 2015, in which they assessed LC in *post-mortem* brains both by histological and 7-T-MRI analysis. They observed that the highest intensity detected by MR occurred at the same level of LC NA complex as assessed by immunohistochemistry for tyrosine beta-hydroxylase [46]. These two studies had been performed by MR scans with different magnetic fields (3 T and 7 T), and in the majority of studies on LC-MRI, a 3 T apparatus has been used; Priovolous et al. have recently shown that data obtained with a 7 T MR scan are highly superimposable with the ones registered with a 3 T scan. Thus, the anatomical confirmation of T1-weighted MRI imaging provided by Keren et al. (2015) may apply to LC-MRI assessment, independently from the field of the MR machine used [47].

Moreover, it has been proposed that LC-MRI may have a good reproducibility, as elegantly shown by Langley and colleagues, which compared LC volume estimation by two different types of MRI scan in eleven subjects [48]. Furthermore, recently Tona and colleagues quantified the test-retest reliability both between different scans and between different operators and they observed a good stability of measured LC intensity and a low variability between different operators [49].

### The Source of LC Signal

The source of LC hyperintensity at MRI is still a matter of debate. The earliest studies, performed at the beginning of 2000s, profited from fast spin echo (FSE)/turbo spin echo (TSE) or gradient echo (GRE) sequences [16, 50–53], according to the hypothesis that NM was the sole responsible for LC signal; in fact, as said, NM binds iron, and this was believed to significantly contribute to T1-shortening effect. Several studies on LC were performed using these sequences in the following years (see Table 1). Such an approach was chosen on the basis of former studies on the SN; since NM accumulates also in SNpc, SN has been extensively evaluated by using the MRI sequences similar to the ones used for LC [54].

Nonetheless, in 2015, Keren and colleagues suggested that a potential source of hyperintense signal was not only the paramagnetic property of NM but rather mainly the magnetization transfer (MT) effect [46]. MT is a MR phenomenon that takes place when magnetization pulses are applied to structures where protons exist in three different states: bounded to macromolecules, in free water, and as a layer between the two latter states. Broadly speaking, MT signal can be obtained thanks to the different frequencies of stimulation that different protonic states have and, in line with this, specific sequences with MT preparation have been developed [55]. In brain imaging, such a technique is widely used both in physiological and pathological conditions, thanks to its ability to increase the contrast between the examined structures and the background [56].

According to Keren and colleagues, the LC-related signal observed by authors using T1-weighted sequences could be even attributed to an “incidental” MT effect, since the high number of radiofrequency pulses in a 2D-FSE scan results in off-resonance magnetization saturation [46, 57].

## LC-MRI in Healthy Subjects (Table 1)

LC development begins in the fetal brain and continues up to late post-natal stages [58], even though remodeling of LC can occur also at later stages [59]. Along adulthood, LC does not undergo major macroscopic changes, except for NM intracellular accumulation, which starts during fetal life and reaches its maximum in the 6th–7th decade [21]. In the past, it had been proposed the occurrence of a reduction of NA cells in LC in healthy elderly subjects compared with younger ones [60]. However, recent stereological analysis (i.e., precise cell-count in post-mortem brain samples) excluded the occurrence a “physiological” degeneration of LC, as it showed a stable NA cells count from adulthood to aging, even when analyzing the brain of oldest subjects [19••].

LC-MRI studies reported results partly in agreement with these observations. In the first study performed in healthy subjects, Shibata and colleagues observed a progressive increase of LC Contrast-Ratio (LC-CR, i.e., the ratio between LC intensity and *tegmentum pontis* one, the latter being considered as a reference region) in parallel with age in young subjects, which reached a *plateau* in adults and then decreased in elderlies. Such a tendency was not linear, but it fitted into an inverted U shaped curve [53]. Other, more recent MRI studies showed an increased LC intensity in elderly subjects [61–63] but in those reports, the authors compared “young” with “elderly” subjects while they did not specifically analyze the LC features along the whole lifespan. In 2019, Liu et al. performed a large analysis on 605 healthy subjects, aged 18–88, and showed an initial increase of LC-CR with age, which reaches its peak in the 6–7th decades and then it decreases in elderly subjects; although a significant reduction of LC signal was not observed in elderlies, a negative correlation between LC rostral third intensity and aging was found [64•]. Intriguingly, variation of LC intensity has been related to cognitive performances in healthy subjects. A higher LC intensity in healthy elderlies was observed to be associated with better cognitive reserve [61] and better emotional memory [65]; it is noteworthy that in one study, such a relationship was found also in young subjects [66]. The variability of the intensity signal in the rostral part of LC has been linked to memory [63] and global cognitive performances [67].

## LC-MRI in Parkinson’s Disease and Other Parkinsonisms (Table 1)

LC degeneration is an early pathological feature of PD, according to Braak et al. [9] who clearly demonstrated that alpha-synuclein accumulation occurs in the LC years before its accumulation at the level of the SNpc. In PD, LC impairment has been put in relation with a variety of non-motor symptoms of PD, ranging from psychiatric alterations to sleep disruption (especially REM behavior disorder (RBD)), to dysautonomic and cognitive impairment [68].

In PD, several authors have shown the occurrence of significant reduction of LC-MRI intensity [16, 69–76]. Noteworthy, and in line with post-mortem pathological observations [13], in PD patients, LC-MRI alteration is even more severe than the SN-MRI one [72, 73]. Even though LC-CR did not show to be correlated with PD disease severity [69, 71, 72], it may be theoretically useful for identifying patients suffering from non-motor symptoms, either as comorbidities in PD patients or as prodromal symptoms of the disease. RBD can occur either alone (idiopathic RBD, iRBD) or after the clinical onset of PD (PD-RBD); LC signal has been shown to be significantly reduced in iRBD [77] and even more in PD-RBD patients [74, 78]. Noteworthy, Sommerauer et al. showed that in PD-RBD patients, LC signal was lower not only compared with HC but also with PD patients lacking RBD [74]. Remarkably, Wang et al. recently found that PD patients suffering from depression showed a more severe alteration of LC signal than those without psychiatric comorbidities; moreover, they observed a significant inverse correlation between LC intensity and depression severity [75]. Such a strict correlation with LC-MRI features was not observed for cognitive and dysautonomic symptoms. In fact, in PD patients suffering from mild cognitive impairment (PD-MCI), LC intensity was reduced compared to HC, but there were no differences between cognitively unimpaired PD patients and PD-MCI ones [74, 76]. Similar results were observed in parkinsonian subjects suffering from dysautonomic dysfunctions [74]. Interestingly, the relationship between LC and autonomic system has been assessed in two further studies; in 2013, Matsuura and colleagues observed that in patients suffering from multi-systemic atrophy (MSA), an atypical parkinsonism type which is very often associated with autonomic system involvement, LC-CR was significantly lower than in HC even in patients lacking a concomitant significant SN-MRI alteration [72]. In 2017, Mather et al. assessed the correlation between LC intensity and high-frequency heart rate variability (HF-HRV) in healthy subjects, the latter being considered as an index of parasympathetic system activity; they observed that LC intensity negatively correlated with HF-HRV and interpreted such a result as a proof of the modulation that LC exerts on the parasympathetic system [79].

## LC-MRI in Alzheimer’s Disease (Table 1)

The occurrence of LC degeneration in AD is a well-established feature of this disorder. Remarkably, as said, LC is the first structure to show AD pathological alterations—namely pTAU protein—already years before the first occurrence of memory complaint [8], and LC shows a significant neuronal loss already in the prodromal stage of AD [10••]. In particular, the latter observation has been obtained by stereological post-mortem analysis, confirming previous evidences [11, 12, 80]. Nonetheless, thus far only few studies have explored LC-MRI features in AD patients. The first ones, which were performed in a limited number of subjects, showed a reduction of LC signal in AD dementia (ADD) patients compared to age-matched healthy controls [81, 82] except for one, which, however, analyzed only a very few patients [83]. In 2019, Betts et al. observed a reduction of LC-MRI intensity in ADD patients and they found that LC-CR correlates with Aβ42/Aβ40 ratio measured in the cerebrospinal fluid (CSF); nonetheless, they did not observe any significant difference between HC and subjects with mild cognitive impairment (MCI) [84•]. An alteration of LC in AD was found also by Olivieri et al. in 2019, which further extended this observation showing a similar degree of LC-CR alteration in typical and atypical ADD [85].

## LC-MRI in Other Neurological Diseases and Psychiatric Disorders

LC impairment has been suggested to play a role also in other neurological diseases, such as migraine [86] and epilepsy [87], as well as in psychiatric disorders [88, 89]. While no studies on LC-MRI have been yet performed in epileptic patients, in 2020, Gollion and colleagues performed an LC-MRI assessment in patients suffering from migraine with aura and they did not find any difference between patients and controls [90]. Concerning psychiatric disorders, LC intensity was found to be reduced in depression [50, 51], while LC volume was increased in subjects suffering from post-traumatic stress disorders (PTSD) and anxiety disorders [91].

## Discussion

In the last decade, LC-MRI studies have provided quite a large amount of data concerning in vivo LC features in physiological and pathological conditions; nonetheless, as described in the “Introduction” section, it is worth mentioning once again that the source of LC contrast is still not fully clear. LC has been assessed by using T1-weighted sequences, either with or without MT preparation; in the first case, the source of MR signal is considered to be directly related to the paramagnetic properties of NM, while for MT, it is considered to be more related to the high protonic density of LC cells.

In an attempt of solving such a question, in 2019, Watanabe and colleagues performed an MRI study (with both FSE and MT approaches) in animal models; in particular, they compared to control mice the LC-MRI scans of two different transgenic mice models: one which was knock-out (KO) for Ear2 gene, a mutation that causes LC agenesis, and one which was KO for dopamine beta-hydroxylase (DBH) gene (the key enzyme for NE synthesis) and thus devoid of NM. They did not observe any LC signal in Ear2-KO mice, while in DBH-KO ones, the signal was detectable and similar to control animals. Since the absence of NM did not hinder LC assessment, these authors hypothesized that MT is the real source of LC signal, rather than NM [92].

According to these findings, some authors supported the latter hypothesis concerning MT and LC [46, 47]; nonetheless, evidences obtained in several LC-MRI studies are hard to interpret with such a view. In particular, LC-CR was found to increase along the life span, with the highest signal observed in elderlies [63, 64•]. This trend could be easily explained considering NM accumulation within LC cells during aging [21] and NM itself as the source of LC intensity, while other interpretations regarding MT-related LC signal variations would be too speculative. Since understating the real source of LC-MRI signal is fundamental for result interpretation, further studies are needed to better clarify this aspect.

In healthy subjects, LC signal shows high inter-subjects variability, an observation largely in agreement with anatomical data [20, 93], and it is independent from age. Interestingly, such variability has been related to cognitive performances [66] and autonomic functions [79]; while in elderlies this relation might herald a latent pathological process (see below), in younger subjects, it might be related to other, not pathological, aspects; LC, similar to other monoaminergic nuclei in the brainstem, is known to undergo neuronal plasticity, in particular in response to hormonal and physical stimulation [94–96]. Thus, such variability may be interpreted not only on the basis of individual characteristics but also as a morphological counterpart of functional modification.

As said, the correlation between memory performance and LC signal in the elderlies [61, 63, 97] could reflect the occurrence of degenerative phenomena. NA is a key modulator of learning processes; thus, the alteration of LC-NA system could hamper memory formation [10••]. Interestingly, studies on HC revealed that LC intensity reduction during aging occurs in the rostral third of LC [63, 97], the same part of LC which projects to hippocampal structures [98] and is more susceptible to AD-related pathology [19••]. Considering that AD incidence increases with age, an alteration of LC-MRI may represent an initial sign of a latent degenerative process in the elderlies, before the occurrence of the first clinical signs.

In line with this, some authors showed that LC signal is reduced in patients suffering from AD when compared to HC, although only few LC-MRI studies, in limited number of patients, have been performed in this disease.

On the other hand, LC-MRI failed to distinguish MCI patients from HC. Since histological studies clearly showed that LC degeneration already occurs in MCI patients [99], the absence of significant differences may be due to study limitations, such as the low number of included subjects. Future studies should focus on detecting LC alterations in early stages of AD pathology, with the aim of developing an LC-related early biomarker.

Finally, it is worth noting that Betts and colleagues observed a relation between CSF amyloid and LC-CR, in line with preclinical data. Indeed in animal model of AD, LC damage has been associated to increased amyloid burden [29, 33], in parallel with a more severe neuroinflammation [30] and to worse neurovascular pathology [31], and neuropathological *post-mortem* data in humans support these observations [10, 100]. Since neuroinflammatory, amyloid and vascular burdens can be evaluated in vivo by specific neuroimaging approaches (e.g., TSPO-specific PET tracer for neuroinflammation [101], amyloid PET and specific MRI sequences for amyloid and vascular alterations, respectively), LC-MRI represents a very interesting opportunity to assess the abovementioned correlations in vivo also in AD patients.

In PD, LC-MRI showed to be able to distinguish patients from HC (see Table 1); LC-MRI signal loss can be found already at early PD stages, and its involvement is even more severe than that observed in the SN [69, 72, 73] in line with histological post-mortem data [13–15]. Interestingly, Isaias and colleagues recently showed in PD patients that LC signal correlates with SNpc signal and Dopamine Transporter (DAT) binding at SPECT DAT scan [73]. It could be hypothesized that patient suffering from a more severe LC-NA degeneration may undergo a greater nigrostriatal impairment. This would be in line with preclinical studies clearly showing that LC lesion leads to a more severe damage of the nigro-striatal DA pathways [39–44].

Moreover, LC-MRI may be potentially used in the future for early diagnosis of PD, already in prodromal stages of the disease. It is worth noting that, since LC-MRI could detect LC impairment in patients suffering from RBD [77, 78] and late onset depression [50, 74], it may be used to assess the risk of developing PD in subjects suffering only from prodromal symptoms. However, several more studies are needed to confirm the specificity of these alterations, by comparing LC-MRI in patients who will develop PD at follow-up with those experiencing only isolated late-onset depression.

## Conclusion and Future Perspective

LC-MRI is a promising technique, which allows the in vivo assessment of LC both in physiological and pathological conditions. This may represent a useful indirect tool to improve our knowledge on the pathogenesis of neurodegenerative diseases; this could be particularly true for AD, for which LC-NA impairment is receiving growing attention. Moreover, LC-MRI is a non-invasive and reproducible assay, thus having the potential to become a new biomarker for degenerative diseases, as recently proposed also by ad-hoc expert consensus [23••]. The possibility of evaluating its integrity may lead to the development of new therapeutic strategies targeting the LC-NA system itself. Indeed, LC degeneration has been linked to increased neuroinflammation and neurovascular disfunction in AD [31, 32], while the administration of NA synthetic precursors (e.g., Threo-DOPS) or NA receptor-agonists has been shown to improve cognitive impairment in AD animal model [102]. Finally, LC-MRI may be used in the future to assess the NA system also in pathological conditions other than NDD, providing a more comprehensive understanding of LC-NA pathophysiology.
